# Treatment of Pneumonia

**Published:** 1907-07

**Authors:** 


### TREATMENT OF PNEUMONIA.

Barr believes that in the treatment of pneumonia the temperature affords a reasonable point of attack, as he strongly favors an increased dissipation of heat. He suggests that, when possible, the patient should have a warm bath, with plenty o soap, or if this be impracticable, he should be well washed, and the action of the skin encouraged by the administration of a diaphoretic. Should the temperature rise as high as 104 F. the sooner a large ice bag is applied to the abdomen the better. Promptitude of action in the early stage, he claims, often arrests the spread of the disease and may even arrest its further progress. With regard to venesection, he has no doubt that in some plethoric cases it does good, but it never should be adopted after the first forty-eight hours, and never at any time if the pulse is small, feeble and compressible. Bleeding, he says, should not be performed on the very young, the old or the debilitated. After bleeding, the patient should abstain from fluid for several hours. He urges particularly the need of good ventilation. The sick room should be the best in the house and, if possible, should contain at least 1000 cubic feet of air for each occupant. The whole air of the room should be changed three times every hour. The administration of oxygen in cases where the sick room ventilation is poor and insufficient he believes to be unscientific, irrational, and absurd. The temperature of the room should be maintained at from 60 F. to 65 F. At this temperature, Barr says, the air is not saturated with moisture; it is capable, therefore, of holding the patient's exhalations, and there is not much danger of him drowning in his own excretions. Speaking of the bronchitis kettle, he says that the man who invented it should have been hanged, as he thinks death from strangulation would be a just punishment for the number of deaths from drowning which his appliance has caused. The bed clothing of the patient should be sufficiently light to allow free escape for the evaporation of the sweat.—The room should be well lighted and cheerful.—Med. Press and Circular, London, May 22nd, 1907.

